# The positive and negative impact of cancer on daily life > 2 years after diagnosis: Results from a survey among 5,710 patients with various cancer types

**DOI:** 10.1007/s11764-025-01870-w

**Published:** 2025-07-17

**Authors:** Floortje Mols, Noortje van Willegen, Dagna Lek, Vivian Engelen

**Affiliations:** 1 CoRPS - Center of Research On Psychological Disorders and Somatic Diseases, Department of Medical and Clinical Psychology, Tilburg University, PO Box 90153, 5000 LE Tilburg, the Netherlands; 2 Department of Research & Development, Netherlands Comprehensive Cancer Organisation (IKNL), Utrecht, The Netherlands; 3 Dutch Federation of Cancer Patients Organizations, Nederlandse Federatie Van Kankerpatiëntenorganisaties (NFK), Utrecht, The Netherlands

**Keywords:** Cancer, Oncology, Survivorship, Patient-reported outcomes

## Abstract

**Purpose:**

This study aimed to understand the positive and negative long-term impact of cancer and its treatment on survivors more than two years post-diagnosis.

**Methods:**

The Dutch Federation of Cancer Patient Organisations (NFK), with input from patient representatives and researchers, created a national online survey. It was distributed via email, websites, and social media to patient organization members, with additional promotion by partner organizations. Participants shared their experiences regarding the physical and psychological effects of cancer on daily life.

**Results:**

A total of 5,710 cancer survivors participated. Of these, 73% felt proud of how they coped, 66% reported that cancer reshaped their priorities, 44% saw new opportunities, and 35% enjoyed life more. However, 58% could not be worry-free, and 49% struggled to resume life. Long-term symptoms were common: 55% experienced both physical and psychological symptoms, 33% reported only physical symptoms, and 2% only psychological. The most frequent physical symptoms included fatigue (56%), reduced fitness (47%), neuropathy (32%), sexual issues (25%), and sleep disturbances (24%). Common psychological symptoms were memory/concentration issues (32%), acceptance struggles (19%), depressive feelings (19%), planning issues (18%), and anxiety (16%).

**Conclusion:**

Cancer survivors often face persistent physical and psychological symptoms, with cancer continuing to affect their lives significantly.

**Implications for cancer survivors:**

Greater awareness of these long-term consequences among healthcare providers and patients is essential.

## Introduction

The number of cancer survivors (i.e. everyone diagnosed with cancer and still alive) is steadily increasing due to the ageing of the population and advances in early detection and treatment. According to a recent study, the population of cancer survivors in the United States alone has surpassed 16.9 million [1]. While this is a significant achievement, cancer survivors often face long-term physical, psychological, and social consequences because of both the disease and its treatment. These include physical problems like chronic pain, fatigue, chemotherapy-induced peripheral neuropathy, in addition to psychological problems such as anxiety, depression, and fear of cancer recurrence [2, 3]. With respect to physical problems, a recent publication concluded that cancer related fatigue is a prevalent and distressing problem for a significant proportion of survivors during active treatment, but also in the survivorship phase [4]. Regarding psychological problems, one study found that anxiety levels remain elevated post-treatment due to concerns about cancer recurrence and reduced medical consultations, while depression rates vary depending on cancer type and stage of treatment [5]. Physical and psychological effects can persist for years, affecting survivors'overall quality of life [6, 7]. Furthermore, many survivors experience difficulties with the impact cancer (still) has on their lives and on what is important in their life [8]. On this latter topic, not much is known while, as the population of cancer survivors grows, addressing these survivorship issues has become a critical component of comprehensive cancer care.

Cancer patient organizations play a crucial role in supporting individuals affected by cancer and helping them navigate the physical and psychological challenges associated with the disease and its treatment. Also, they play an important role in helping patients get back on their feet after cancer. For instance, patient organizations empower individuals by providing reliable information about their diagnosis, treatment options, and survivorship care. In addition, they often advocate for patients'rights and access to care. For instance, they work to improve healthcare policies, reduce disparities in cancer care, and ensure that patients have access to necessary treatments, regardless of socioeconomic status. They also raise awareness about the long-term physical and psychological consequences of cancer treatment. Furthermore, these organizations play a vital role in facilitating the reintegration of survivors into daily life, for instance through facilitating peer-to-peer contact and public awareness campaigns. Overall, they act as a bridge between patients and the healthcare system. To do so, they need information on the needs and experiences of cancer survivors.

The Dutch Federation of Cancer Patient Organisations (NFK in Dutch) currently represents 19 cancer patient organisations. NFK has four main goals: appropriate care in the right hospital; equal access to innovative treatments and diagnostics; early detection of cancer in (high) risk groups; and living a good live with cancer. The NFK designs and distributes several questionnaires each year to explore the needs and experiences of cancer patients on diverse subjects regarding their main goals. The findings from these questionnaires enable NFK to address specific gaps, shortcomings or needs from the perspective of the patient, and help them advocate and work on specific solutions together with healthcare professionals.

The aim of the current study was related to their last goal, namely, to get a better understanding of cancer patients’ needs and experiences a longer time after diagnosis. This included getting a clear view on the physical and psychological consequences of cancer or its treatment, and especially how this affects life, both positively and negatively. By having a clear view on which effects of cancer are experienced by this large and diverse group and when they occur, the NFK can advocate more specifically for the interests of people living with or after cancer.

## Methods

### Study design and participants

For this study, an online questionnaire was designed to examine patients’ needs and experiences. This questionnaire was filled out by individuals living in the Netherlands that were diagnosed with cancer at least 2 years ago. Data were collected through the programme Survalyzer. The survey was open for two weeks in the fall of 2023 through the NFK (https://doneerjeervaring.nl/) and various social media (sponsored and organic posts on Facebook and Instagram; LinkedIn and Twitter/X only organic posts). Also, the cancer patient organisations united within NFK send their members and donors an e-mail with an invitation to complete the questionnaire, and they also used various social media to recruit respondents. In addition, members of the Donate Your Experience (DJE) panel received an e-mail invitation for the questionnaire. Finally, partner organisations such as the Dutch Cancer Society, Kanker.nl, IPSO Centres for living with or after cancer, and some hospitals through their own channels, asked people to complete it. While formal medical verification was not feasible due to the anonymous and large-scale nature of the survey, eligibility criteria and the use of established networks help ensure the reliability of the sample.

All participants were informed about privacy policies in accordance with the General Data Protection Regulation (EU) and all gave informed consent. Since this was an online questionnaire without an intervention, the Medical Research Involving Human Subjects Act (WMO) did not apply, and therefore ethical approval was not required. No monetary or material incentives were provided for participation.

### Questionnaire

The questionnaire was developed by a group of patient representatives from five different cancer patient organisations, a patient advocate and researcher from the NFK, a consultant from the.

Netherlands Comprehensive Cancer Organisation (IKNL), a researcher from the PROFILES-registry, and a communication advisor from the Dutch Cancer Society. The questionnaire was reviewed for content and face validity through a continuous process of reflection within the team, resulting in an iterative version of the questionnaire.

Participants self-reported their type of cancer by selecting it from a predefined list. Also, they filled out in what year they were diagnosed. Patients who indicated that they were diagnosed less than 2 years ago were excluded from filling in the questionnaire any further. The questionnaire included demographic variables (e.g., age, gender, education level), cancer-related information (e.g., type and year of diagnosis, treatment status), and a series of structured and open-ended questions on physical and psychological consequences and the broader impact of cancer on life. Key closed-ended questions included:

- 'What physical or psychological symptoms have you experienced in the past 3 months due to cancer or its treatment?'(checkbox list of predefined symptoms)
- 'What is the influence of cancer on your life?'(multiple options, e.g., “I enjoy life more,” “I struggle to resume life”)
- 'How many symptoms have you experienced?'(numeric input)

Items were either binary (yes/no), multiple-response (checkboxes), or categorical with predefined options. Open-ended responses were collected to enrich the structured data but were not subject to formal qualitative analysis. In this manuscript, they are included solely to illustrate and contextualize quantitative findings. It was not possible to skip questions, except for the open-ended ones. Respondents were able to stop and save their answers and complete the survey at some other point in time. The questionnaire was anonymous, and the average completion time was estimated at 15 min.

### Statistical analysis

Categorical variables were presented as numbers and percentages. Differences between subgroups based on gender, age at diagnosis, education level, time since diagnosis, disease phase, treatment phase, and cancer type were compared by Pearson chi-square test for categorical variables. Where applicable, this excludes the response categories: ‘don't know/not applicable’ and ‘other’. This may cause the percentages to be slightly higher. Statistical analyses were performed using IBM SPSS Statistics version 28. Only outcomes where there was a relevant and significant difference of at least 10% with a p-value < 0.05 were included in this paper.

## Results

### Participants

**Fig. 1:**
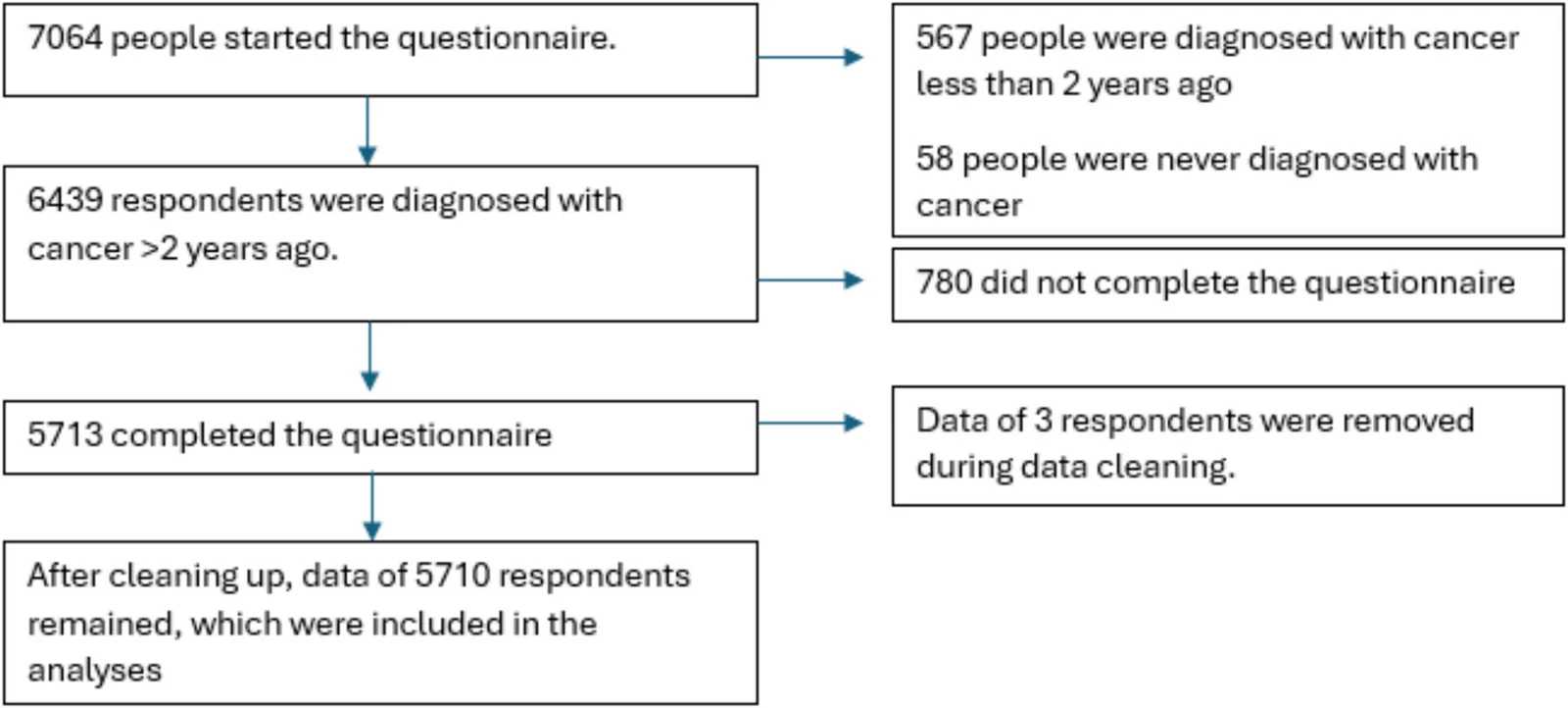
Flow chart

A total of 5710 respondents fully completed the questionnaire (Fig. 1, Table 1). More than half were women (59%), and highly educated (53%). Two thirds were currently ≥ 60 years (67%) and the most common diagnoses were breast cancer (30%), haematological cancer (25%) and prostate cancer (17%). Sixty-one percent were < 60 when they heard they had cancer, and for 40% it was between 5–10 years ago. At the time of completing the questionnaire, 26% of respondents were undergoing cancer treatment, and nearly two-thirds (64%) reported being cured or expecting to be cured. Forty-four percent were treated at a top clinical cancer hospital.

**Table 1:** Sociodemographic and clinical characteristics of participants

		N	%
Gender	Male	2353	41
	Female	3346	59
	Other	11	0
Education level	Low	629	11
	Medium	1962	34
	High	3016	53
	I prefer not to say/different	103	2
Age at time of questionnaire	≤ 18 years	1	0
	19 and 39 years	182	3
	40 and 59 years	1696	30
	60 and 74 years	2758	48
	≥ 75 years	1073	19
Cancer type	Anal cancer	31	1
	Bladder or kidney cancer	166	3
	Blood or lymphatic cancer	1433	25
	Breast cancer	1706	30
	Colorectal cancer	206	4
	Gynaecological cancer	265	5
	Brain tumour	65	1
	Head/neck cancer	111	2
	Lung cancer	194	3
	Gastric or oesophageal cancer	169	3
	Melanoma or skin cancer	89	2
	Prostate cancer	977	17
	Sarcoma	62	1
	Thyroid cancer	35	1
	Testicular cancer	53	1
	Other	148	3
Age at diagnosis	≤ 18 year	41	1
	19–39 years	590	10
	40–59 years	2832	50
	60–74 years	2004	35
	≥ 75 years	243	4
Time since diagnosis	> 2 to 5 years ago	1850	32
	5–10 years ago	2289	40
	> 10 years ago	1571	28
Currently in treatment	Yes	1494	26
	No, but still follow-ups	2786	49
	No, also no follow-ups	1066	19
	No, never had, wait and see	139	2
	No, had treatment before, wait and see now	225	4
Disease phase	I (probably) don’t have cancer anymore	3497	61
	I have cancer and will (probably) get better	167	3
	I have cancer and will (probably) not get better	884	16
	I have a chronic cancer type	912	16
	I have cancer and don’t know if I will get better	250	4
Hospital	University medical centre/cancer hospital	1754	31
	Top clinical hospital	2492	44
	General hospital	1261	22
	Other	203	2

At the time of diagnosis, a significantly higher percentage of women (77%, n = 2586) were under 60 years of age compared to men (37%, n = 870). In addition, they were significantly more likely to say they were probably (to be) cured (75%, n = 2462) than men (55%, n = 1192). Among those diagnosed before the age of 60, 36% (n = 1242) had been diagnosed > 10 years ago, compared to 15% (n = 329) of respondents aged ≥ 60. Additionally, respondents diagnosed before the age of 60 were significantly more likely to report they were probably (to be) cured (73%, n = 2460) compared to those diagnosed at ≥ 60 years (58%, n = 1204).

### Physical and psychological symptoms due to cancer (treatment)

In the past 3 months, 55% (n = 3122) of respondents experienced both physical and psychological symptoms due to cancer and its treatment, 33% (n = 1859) only reported physical symptoms, 2% (n = 101) only psychological, and 11% (n = 628) reported no symptoms. The number of symptoms among respondents varied widely. On average, respondents reported 5.9 symptoms (SD = 3.8; range = 0–22). Of these, 55% (n = 2735) reported 1–5 symptoms, 33% (n = 1612) had 6–10 symptoms, 10% (n = 498) had 11–15 symptoms, and 2% (n = 104) had 16–22 symptoms.

Women (64%, n = 2150) were significantly more likely than men (41%, n = 967) to report both physical and psychological symptoms. Respondents diagnosed under 60 years of age (62%, n = 2161) were significantly more likely to report these symptoms compared to older individuals (43%, n = 961). Those currently in treatment (65%, n = 970) were also significantly more likely to report both types of symptoms than those not in treatment (51%, n = 2152).

Respondents diagnosed 2–5 years ago (59%, n = 1094) and 5–10 years ago (57%, n = 1298) reported physical and psychological symptoms significantly more often than those diagnosed > 10 years ago (47%, n = 730). Among the latter group, 15% (n = 234) reported no symptoms, compared to 10% for both the 5–10 years group (n = 218) and the 2–5 years group (n = 176). While not statistically significant and relevant, these differences are noteworthy.

### Physical and psychological symptoms due to cancer (treatment) in detail

In the past 3 months, the most common physical symptoms due to cancer and its treatment were fatigue (56%), reduced physical fitness (47%), neuropathy (32%), sexual problems/impotence (25%), and sleep problems (24%). Thirteen percent experienced no physical symptoms. The most common psychological symptoms were memory or concentration problems (32%), issues accepting cancer (19%), depressive feelings (19%), planning difficulties (18%), and anxiety (16%). Forty-four percent experienced no psychological symptoms.

Women were significantly more likely than men to report: fatigue (64%, n = 2130 vs. 46%, n = 1069), lymphoedema (22%, n = 729 vs. 7%, n = 174), pain (26%, n = 880 vs. 11%, n = 265), neuropathy (38%, n = 1262 vs. 24%, n = 567), sleep problems (30%, n = 1002 vs. 16%, n = 384), menopausal or hormonal symptoms (25%, n = 827 vs. 7%, n = 155), osteoporosis (16%, n = 519 vs. 6%, n = 130), anxiety (21%, n = 712 vs. 8%, n = 196), problems with changed appearance (17%, n = 574 vs. 4%, n = 85), memory or concentration problems (40%, n = 1346 vs. 20%, n = 474), and planning difficulties (23%, n = 773 vs. 11%, n = 263). Men significantly more often suffer from sexual problems (37%, n = 876 vs. 17%, n = 556).

Respondents < 60 years old at diagnosis were significantly more likely to report symptoms due to cancer and its treatment than those aged ≥ 60: fatigue (63%, n = 2181 vs. 46%, n = 1026), lymphoedema (20%, n = 692 vs. 10%, n = 213), pain (26%, n = 881 vs. 12%, n = 266), menopausal or hormonal symptoms (24%, n = 840 vs. 6%, n = 142), problems with changed appearance (16%, n = 539 vs. 5%, n = 120), memory or concentration problems (40%, n = 1390 vs. 19%, n = 434), and planning difficulties (24%, n = 825 vs. 10%, n = 212).

Fatigue (60%, n = 1101), memory or concentration problems (35%, n = 636), problems accepting (the consequences of) cancer (25%, n = 468) and menopausal or hormonal symptoms (22%, n = 406) are significantly more often reported by respondents who are between 2–5 years after diagnosis than respondents who are > 10 years after diagnosis (49%, n = 770; 25%, n = 389; 14%, n = 213; 11%, n = 170) respectively (Table 2). Additionally, those > 10 years after diagnosis are significantly more likely to report no psychological symptoms (53%, n = 822 vs. 39%, n = 726).

**Table 2:** Physical and psychological symptoms that patients suffer from due to (the treatment of) cancer

*Did you suffer from physical or psychological symptoms due to (the treatment of) cancer?* *In the past 3 months*	Time since diagnosis % Yes			Disease phase % Yes		Currently in treatment? % Yes	
	*Between 2–5 years n* = *1850*	*Between 5–10 years* *n* = *2289*	> *10 years* *n* = *1571*	*(Probably) better* *n* = *3664*	*(Probably) not better* *n* = *1796*	*Yes* *n* = *1494*	*No* *(n* = *4216)*
Fatigue	60*	59	**49**	**52**	66*	70*	**52**
Reduced physical fitness	50	46	44	**42**	57*	60*	**42**
Memory or concentration problems	35*	35	**25**	32	33	40*	**29**
Neuropathy	30	36	29	31	36	39	30
Sexual problems/impotence	27	26	22	25	25	31	23
Sleep problems	27	26	20	23	28	32*	**22**
Difficulty accepting cancer	25*	19	**14**	19	20	24	18
Feelings of gloom or depression	22	19	16	17	23	26	17
Problems with planning or keeping an overview	22	19	13	19	18	22	17
Pain	21	22	17	21	20	26	18
Fear	20	16	11	17	15	19	15
Shortness of breath	18	18	17	15	23	24	16
Stomach or intestinal problems	19	19	18	16	23	25	16
Problems with movement	18	18	18	16	21	25*	**15**
Menopausal or hormonal symptoms	22*	18	**11**	21	11	28*	**14**
Lymphoedema	15	17	17	19	10	17	16
Problems with eating, drinking or weight	17	17	13	16	16	21	14
Problems with changed appearance	13	12	9	13	10	14	11
Doubts about (the meaning of) life	12	9	9	9	11	12	9
Oral or dental problems	11	12	11	11	13	14	11
Incontinence	11	12	12	11	12	13	11
Skin problems	9	11	12	8	16	16	9
Osteoporosis	8	13	14	11	12	17	10
Problems with vision, hearing or talking	8	8	9	8	10	10	8
Cardiac problems	5	7	11	8	7	7	8
Problems with blood vessels	4	5	6	4	5	5	4
Fertility problems	2	2	2	3	1	1	2
No physical symptoms	11	12	16	15	10	**4**	16*
No psychological symptoms	**39**	41	53*	45	42	**35**	47*
No symptoms	10	10	15	13	8	**4**	14*

^*^ = some symptoms are significantly (p < 0.05 and a difference of ≥ 10%) more common among those with certain background characteristics. These percentages are in bold

Fatigue (66%, n = 1180) and reduced physical fitness (57%, n = 1019) were significantly more common in respondents who will (probably) not get better compared to those who will or are (probably) getting better (52%, n = 1890; 42%, n = 1520; Table 2). Respondents currently in treatment reported significantly more fatigue (70%, n = 1037), reduced physical fitness (60%, n = 893), memory problems (40%, n = 594), sleep problems (32%, n = 479), problems with movement (25%, n = 367), and menopausal or hormonal symptoms (28%, n = 411) than those not undergoing treatment at the time of survey completion (52%, n = 2170; 42%, n = 1770; 29%, n = 1230; 22%, n = 910; 15%, n = 643; 14%, n = 571) (Table 2). Those not receiving treatment were significantly more likely to report no physical and/or psychological symptoms (14%, 574) versus those receiving treatment (4%, n = 54).

For all cancer types, fatigue and reduced physical fitness are among the top 5 most common symptoms (Table 3, top 5 is in bold). Neuropathy is also frequently in the top 5, while other symptoms vary by cancer type.

**Table 3:** Physical and psychological symptoms that patients suffer from due to (the treatment of) cancer

Did you experience physical or psychological symptoms due to (the treatment of) cancer? In the past 3 months	Anal cancer N = 31	Bladder or kidney cancer N = 166	Blood or lymphatic cancer N = 1433	Breast cancer N = 1706	Colorectal cancer N = 206	Gynaecological cancer N = 265	Brain cancer N = 65	Head and neck cancer N = 111	Lung cancer N = 194	Gastric or oesophageal cancer N = 169	Melanoma or skin cancer N = 89	Prostate cancer N = 977	Sarcoma N = 62	Thyroid cancer N = 35	Testicular cancer N = 53
Fatigue	**45**	**39**	**66**	**64**	**46**	**56**	**72**	**54**	**62**	**50**	**35**	**37**	**48**	**88**	**34**
Reduced physical fitness	**42**	**38**	**55**	**48**	**40**	**45**	**59**	**43**	**66**	**56**	**34**	**35**	**39**	**50**	**26**
Memory or concentration problems	32	16	**32**	**45**	**21**	**34**	**65**	22	**38**	23	**16**	14	**38**	**47**	**25**
Neuropathy	16	**19**	**43**	**41**	**30**	27	31	16	**31**	22	14	12	**34**	21	**32**
Sexual problems/impotence	**48**	**26**	14	20	19	**31**	14	14	13	15	7	**61**	18	15	19
Sleep problems	19	**21**	**22**	33	15	24	**43**	20	29	**28**	14	**16**	20	**35**	6
Difficulty accepting cancer	23	12	17	22	15	25	37	22	27	14	15	**16**	23	**41**	21
Feelings of gloom or depression	13	15	19	21	12	21	37	20	23	16	15	**16**	20	32	21
Problems with planning or keeping an overview	19	9	17	27	11	21	**46**	**12**	21	11	**16**	8	16	32	13
Pain	**42**	14	15	32	10	21	19	27	23	10	**17**	9	**34**	27	13
Fear	23	13	12	23	13	25	26	15	27	10	7	7	16	24	21
Shortness of breath	10	15	**22**	17	10	11	11	22	**49**	27	14	10	18	18	8
Stomach or intestinal problems	**65**	10	**22**	15	**27**	**29**	15	16	17	**44**	9	10	30	27	6
Problems with movement	26	11	19	22	10	15	31	17	26	10	11	11	**31**	32	11
Menopausal or hormonal symptoms	0	1	4	**37**	5	**29**	17	2	7	2	4	14	10	24	4
Lymphoedema	7	4	6	32	6	26	3	17	12	2	**17**	9	21	9	2
Problems with eating, drinking or weight	10	9	10	18	10	14	15	**53**	25	**61**	12	8	16	**41**	8
Problems with changed appearance	10	1	7	21	10	15	12	19	14	4	9	4	16	9	8
Doubts about (the meaning of) life	7	8	10	11	8	15	22	12	13	9	14	5	13	24	11
Oral or dental problems	7	2	14	14	9	9	19	**51**	18	14	8	3	12	18	2
Incontinence	**58**	14	7	5	14	20	8	3	5	7	5	**33**	8	0	0
Skin problems	19	7	17	9	7	8	9	15	12	5	**22**	3	13	21	6
Osteoporosis	13	3	10	20	6	8	5	6	6	8	7	7	15	9	4
Problems with vision, hearing or talking	3	5	10	8	4	6	26	**31**	12	8	11	3	13	12	21
Cardiac problems	13	4	10	8	5	5	6	7	8	8	8	3	8	21	9
Problems with blood vessels	3	7	6	4	3	5	3	6	8	4	2	3	10	0	6
Fertility problems	3	0	1	2	1	8	3	1	0	1	0	1	5	3	**23**
No physical symptoms	3	31	12	8	27	9	0	11	9	7	44	15	20	6	23
No psychological symptoms	45	59	47	31	52	36	12	41	34	54	62	61	38	32	49
No symptoms	3	28	11	7	23	8	0	9	8	6	40	14	15	6	11

^*^ Top 5 is in bold

### The influence of cancer on life

Nearly three-quarters (73%, n = 4174) of respondents felt proud about how they coped with cancer. Two-thirds (66%, n = 3786) reported that cancer made other aspects of life important. However, more than half (58%, n = 3339) said they could never be worry-free again, and nearly half (49%, n = 2791) found it difficult to resume their life after their diagnosis. Almost half (44%; n = 2492) see other possibilities in life because of cancer and a third (35%, n = 1981) enjoys life more than before they had cancer. Over a quarter (28%, n = 1618) struggle with how they want to live their lives because of cancer. Finally, 19% (n = 1090) are no longer preoccupied with cancer.

Respondent; “The moment you are better, everything falls away at once, the visits to the hospital, etc. There are check-ups, but then you suddenly must start living again and trust that ‘everything’ is okay again.”

### The influence of cancer on life in detail

Women and those younger than 60 at diagnosis more often felt proud about how they coped with cancer, they more often see other possibilities in life because of cancer, they stated more often that cancer made other aspects of life more important, and they enjoy life more than before they had cancer (Table 4). However, women and those younger than 60 at diagnosis also more often stated that they could never be worry-free again, that they struggled to resume life after their diagnosis, that they struggle with how they want to live their lives because of cancer. The influence of cancer on life did not differ significantly with respect to education level.

Respondent; “I have become easier, more loving. Also worry less”.

**Table 4:** How did cancer (treatment) impact your life?

	Women	Men N (%)	< 60 years at diagnosis	> 60 at diagnosis
I feel proud of how I coped with cancer	2697 (81)	1468 (63)*	2728 (79)	1446 (65)*
I see other possibilities in life because of cancer	1675 (51)	813 (36) *	1836 (54)	656 (31)*
Cancer made other aspects of life important	2378 (71)	1403 (60)*	2560 (74)	1226 (55)*
I enjoy life more than before I had cancer	1349 (41)	627 (27)*	1447 (42)	534 (24)*
Because of cancer I will never be worry-free again	2237 (67)	1097 (47)*	2216 (64)	1123 (51)*
I found it difficult to resume life after diagnosis	1958 (59)	830 (35) *	2005 (58)	786 (35) *
I struggle with how I want to live my life because of cancer	1096 (33)	519 (22)*	1177 (34)	441 (20)*
I am no longer preoccupied with cancer	476 (14)	607 (26)*	533 (15)	557 (25)*

^*^P < 0.001

Respondents diagnosed with cancer > 10 years ago were significantly more likely (26%, n = 411, p < 0.001) to say they were no longer preoccupied with it, compared to those diagnosed 5–10 years (18%, n = 412) and 2–5 years ago (15%, n = 267). Those diagnosed 2–5 years ago were significantly more likely (36%, n = 651) to struggle with how to live their lives, compared to those diagnosed 5–10 years (29%, n = 653) and over 10 years ago (20%, n = 314, p < 0.001).

Respondents who are (probably) (getting) better are significantly more likely (24%, n = 859) to no longer be preoccupied with cancer compared to those (probably) not (getting) better (11%, n = 195, p < 0.001). They are also significantly more often proud of how they have coped with the disease (78%, n = 2836 vs. 67%, n = 1178, p < 0.001) and more likely to say they enjoy life more (39%, n = 1415) than the group of respondents who are (probably) not (getting) better (28%, n = 505, p < 0.001).

Respondent; “I enjoy the fun things and especially my grandchildren”.

Respondents not currently in treatment were significantly more likely (23%, n = 967) to say they were no longer preoccupied with cancer, than those currently in treatment (8%, n = 123, p < 0.001). Moreover, respondents in treatment were significantly more likely (38%, n = 558) to say they struggle with how they want to live their lives than those not in treatment (26%, n = 1060, p < 001).

## Discussion

In this study of 5,710 individuals diagnosed with cancer at least two years prior, 73% felt proud of how they coped, 67% found life’s other aspects more meaningful, 43% saw new possibilities, and 35% enjoyed life more than before. However, 58% reported they could never be worry-free, and 49% struggled to return to normal. Nineteen percent felt unaffected by cancer's impact. Fifty-five percent reported physical and psychological symptoms, 33% only physical, 2% only psychological, and 11% reported no symptoms. Common physical symptoms included fatigue (56%), reduced fitness (47%), neuropathy (32%), sexual problems (25%), and sleep problems (24%) while psychological symptoms included memory issues (32%), acceptance struggles (19%), depressive feelings (19%), planning difficulties (18%), and anxiety (16%).

This study shows that for many people, cancer has a major impact on their lives, even long after diagnosis. For example, half of the respondents indicated that they found it difficult to resume life again after diagnosis and more than half say they are never carefree. Cancer often means intensive treatments, which can leave traces both physically and psychologically. Learning to continue life with that turns out to be challenging [9–11]. At the same time, for many people, the experience of cancer causes them to value life differently. In addition, almost three quarters were proud of how they coped with cancer, which is a hopeful and encouraging finding.

An overwhelming majority (89%) of respondents experienced at least one or more physical and/or psychological symptoms due to cancer, even > 10 years after diagnosis (85%). Many physical symptoms, like neuropathy and lymphoedema, persist over time, with nearly half still experiencing fatigue after 10 years. These results confirm the outcomes of many other studies on symptoms among cancer survivors [12–15]. However, the prevalence of psychological symptoms seems to decrease over time in our study. As one respondent shared, “In the beginning, I was mostly scared, later I calmed down in this and I now enjoy the little things.” It is encouraging that people learn to cope better and regain confidence over the years, though some still struggle with psychological symptoms like depressive feelings and anxiety. Some studies on this topic report that psychological distress increases in the years after treatment [16, 17]. Others show different trajectories [18, 19]. For instance, a recent study from Portugal identified 3 trajectories of anxiety over time: (1) high scores that increase over time (22%); (2) consistently low levels (64%); (3) mid-range scores at baseline that decrease over time (15%) and 3 trajectories for depressive symptoms over time: (1) high scores that increase over time (21%); (2) mid-range scores at baseline decreasing over time (59%); (3) consistently low scores (20.2%) [19]. The finding that distress and symptoms decline over time since diagnosis is consistent with adaptation theories, where survivors gradually adjust to changes and re-establish life routines. Selection effects may also play a role—those with persistent distress may be less likely to respond. This pattern aligns with findings from Harrington et al. (2010), who documented persistent but gradually diminishing symptom burden in long-term cancer survivors spanning multiple cancer types [12]. Differences between studies can also be caused by differences in measurement instruments used to assess psychological symptoms, the timing of the assessments, and differences in included populations (e.g. age, gender, tumour type). The observed differences by gender and age may reflect both biological and psychosocial processes. For example, women are more likely to report symptoms and psychological distress, potentially due to greater health awareness, emotional expression, or hormonal factors. Younger survivors may face greater disruption to career, identity, and family life, which could contribute to higher distress.

Although many of these findings confirm what is already known, this study contributes valuable patient-centred data from a large, diverse Dutch sample with a high response rate. The high response rate is likely due to the strong engagement of Dutch cancer patients with the NFK and the high trust in affiliated organizations. This may reflect cultural factors such as a high degree of civic engagement, trust in health-related organizations, and openness around cancer survivorship in the Netherlands. This high response rate allows us to get a good picture of people living with or after cancer. It also highlights long-term symptom persistence and the dual positive and negative impacts of survivorship, which are underreported in quantitative literature. However, this study has some weaknesses that need to be mentioned. First, we see that the participating women are a lot younger than the participating men. When interpreting analyses regarding age and gender, this should be considered. Secondly, we see that level of education is not representative for the Netherlands. In this study, 11% are practically, 34% medium and 53% highly educated compared to the 29%, 40% and 30% respectively from Statistics Netherlands. There is thus an underrepresentation of practically educated and an overrepresentation of highly educated cancer survivors. Although the absolute number of practically educated respondents is high (n = 629) which made group analysis possible, this should also be considered when interpreting the results of this study. Next, while our population includes individuals with a variety of cancer types, we acknowledge that these represent biologically and clinically distinct diseases with unique trajectories and consequences. The purpose of our study was not to compare cancer types, but to gain insight into shared long-term experiences across diagnoses. Nevertheless, this heterogeneity is a limitation and should be taken into account when interpreting subgroup differences. Related to that, breast cancer, haematological cancer and prostate cancer are dominant in this study. Other cancer types are much smaller in number. In part, this is logical, as breast and prostate cancer are common. Nevertheless, this should be considered when interpreting the results. Some symptoms are, in fact, clearly related to the cancer type and its treatment. For instance, sexual problems or incontinence among prostate cancer survivors. In addition, we did not collect specific clinical details such as tumour stage, treatment regimen, or recurrence. This limits our ability to adjust findings for those potentially confounding clinical factors. Also, the survey was not based on previously validated scales but was developed collaboratively with patients and experts to ensure content relevance and face validity. This approach prioritizes patient-centred insights but limits comparability with standardized instruments. Another limitation of the survey is that it did not include a specific question on fear of cancer recurrence, despite its relevance to long-term survivorship. While anxiety was addressed generally, future work should include validated fear of cancer recurrence measures. Finally, there may be selection bias. This online questionnaire was publicly distributed. Anyone who felt drawn to the title ‘*Living with or After Cancer: What Is It Like for You?*’ and who was diagnosed with cancer > 2 years ago could complete the questionnaire. People who are not dealing with cancer anymore might be less motivated to participate. Also, the questionnaire could only be completed online which could lead to selection bias. For (older) people who are not so digitally proficient, this may be a barrier to participation. In addition, we recruited participants mainly among the populations of cancer patient organisations. Perhaps these patients are already a bit more aware of their illness than people who are not affiliated with a patient organisation. We made every effort to ensure the title and introduction of this poll were as neutral and unbiased as possible. The introduction included the statement: “Even if cancer does not affect your life now, you help us by filling in the questionnaire.” Additionally, hospitals and organizations like the Dutch Cancer Society helped distribute the questionnaire, enhancing its representativeness. Although we recruited participants via well-established cancer organizations, patient communities, and healthcare partners, all of which primarily reach individuals with a confirmed cancer history, we were not able to medically verify respondents’ cancer diagnoses.

In conclusion, this study showed that cancer survivors still report physical and psychological symptoms long after cancer diagnosis and treatment. Also, cancer still plays an important role in the lives of many survivors. More awareness should be raised on the late consequences of cancer and its treatment on life among health care professionals and patients. Therefore, the results of this study gave rise to several actions. The NFK published the results of this study on their website and shared it via social media. Affiliated cancer patient organizations were also encouraged to share the results. In addition, the results were published in the national media (i.e., newspapers and television) to raise awareness about the long-term effects of cancer. Furthermore, the NFK shared the results with relevant stakeholders, including healthcare professionals and employers, for instance via hospitals, at conferences, and via oncological care networks. Finally, NFK collaborates with cancer patient organizations to initiate projects or activities around the late consequences of cancer. For instance, the data of this study were used in the development of a pilot project which arranges a fixed point of contact in primary care for cancer patients. Also, data from this project were used as a basis to make choices for projects and activities for the goal ‘late effects’ in the Dutch cancer research agenda. Finally, a guide ‘late consequences at work’ has been developed for labour experts that includes this data.

## Data Availability

Data is securely stored and can be viewed at the Dutch Federation of Cancer Patient Organisations (NFK). Restrictions apply to the use of this data, which was used under license for this study.

## Abbreviations

DJE panel: Donate Your Experience
IKNL: Netherlands Comprehensive Cancer Organisation
NFK: Dutch Federation of Cancer Patient Organisations
WMO: Medical Research Involving Human Subjects Act
